# Adolescent idiopathic scoliosis: The mechanobiology of differential growth

**DOI:** 10.1002/jsp2.1115

**Published:** 2020-07-24

**Authors:** Theodoor H. Smit

**Affiliations:** ^1^ Department of Orthopaedic Surgery Amsterdam Movement Sciences, Amsterdam University Medical Centres Amsterdam Netherlands; ^2^ Department of Medical Biology Amsterdam University Medical Centres Amsterdam Netherlands

**Keywords:** adolescent idiopathic scoliosis, differential growth, Hueter‐Volkmann law, intervertebral disc, notochordal cells, tensegrity

## Abstract

Adolescent idiopathic scoliosis (AIS) has been linked to neurological, genetic, hormonal, microbial, and environmental cues. Physically, however, AIS is a structural deformation, hence an adequate theory of etiology must provide an explanation for the forces involved. Earlier, we proposed differential growth as a possible mechanism for the slow, three‐dimensional deformations observed in AIS. In the current perspective paper, the underlying mechanobiology of cells and tissues is explored. The musculoskeletal system is presented as a tensegrity‐like structure, in which the skeletal compressive elements are stabilized by tensile muscles, ligaments, and fasciae. The upright posture of the human spine requires minimal muscular energy, resulting in less compression, and stability than in quadrupeds. Following Hueter‐Volkmann Law, less compression allows for faster growth of vertebrae and intervertebral discs. The substantially larger intervertebral disc height observed in AIS patients suggests high intradiscal pressure, a condition favorable for notochordal cells; this promotes the production of proteoglycans and thereby osmotic pressure. Intradiscal pressure overstrains annulus fibrosus and longitudinal ligaments, which are then no longer able to remodel and grow, and consequently induce differential growth. Intradiscal pressure thus is proposed as the driver of AIS and may therefore be a promising target for prevention and treatment.

## Graphical abstract

Differential growth has been suggested as a physical mechanism for spinal deformations in Adolescent Idiopathic Scoliosis. Here, the underlying mechanobiological mechanisms are explored and it appears that high intradiscal pressure may be responsible for overstraining the annulus fibrosus and longitudinal ligaments, thereby inducing differential growth.

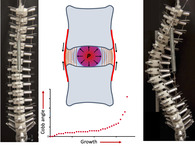

## 1 INTRODUCTION

The human body is a marvel of mechanics. It moves upright in an intrinsically unstable position, it can lift more than its own body weight, it can run for hours, it can perform meticulous tasks like hand writing, and it can throw and shoot with remarkable precision. Intentional motion is controlled by the brain, but there are two principles that underlie the remarkable mechanical functionality of the musculoskeletal system: *functional adaptation* and *tensegrity*. William Roux' concept of Functional Adaptation, published in 1881^1^ and better known as “use it or lose it,” describes the notion that biological organs and tissues are “adapted by making use of it,” that is: reinforced when used and broken down when unused. Trained muscles, tendons, and ligaments indeed increase in size and strength, and atrophy when rested for a longer period of time. The same applies to bone, where Roux' principle implies that trabeculae are aligned along the lines of principal stress (*Wolff's Law* ^2^), resulting in optimal, minimum‐weight structures.^3^, ^4^ D'Arcy Thompson^5^ pointed out that, as a result of functional adaptation, the architecture of an organism reflects the forces it is subjected to, both in healthy and in diseased or traumatized bodies.

**FIGURE 1:**
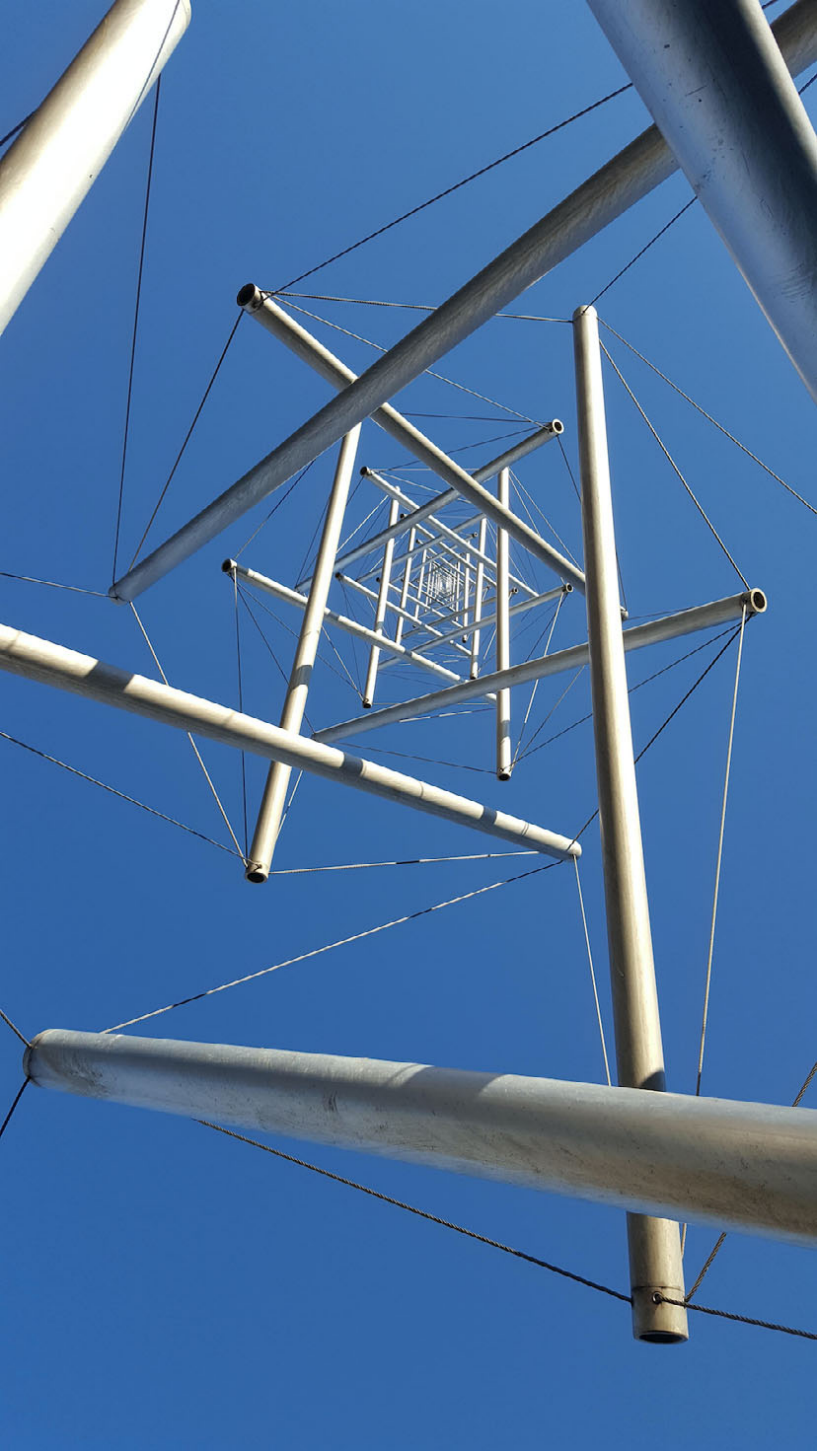
An example of tensegrity: the 18 m high Needle Tower at the Hirshhorn Museum and Sculpture Garden, Washington D.C., designed by Kenneth Snelson. The rods are loaded under pure compression, the wires under pure tension. Picture by Saku Takakusaki

*Tensegrity*, coined by Buckminster Fuller as a short name for tensional integrity,^6^ is a structural principle in which a number of isolated rods are connected to each other by a network of tensile bands (Figure 1). As all cables are fixed to the ends of the rods, the latter are loaded under pure compression, while the former apply pure tension. The stability of the construct increases with higher stiffness and prestress of the tensile elements. An essential property of tensegrity structures is that in all elements the tensile and compressive stresses are balanced and therefore interacting: a change of stress in one element results in a disturbed balance in all other elements, which therefore must adjust their position to restore equilibrium.

Musculoskeletal systems have much in common with tensegrity structures, a concept often referred to as *biotensegrity*.^7^ In this view, bones are the compressive components, while the tensile elements are represented by muscles, tendons, ligaments, and fascia. However, there are also some important differences. One is that bones are not isolated elements: they touch each other in the articular joints where they transfer substantial loads and slide along each other (with the shoulder blades as a notable exception). Also, fibrocartilaginous joints that do not articulate, like the intervertebral discs, undergo substantial mechanical loading.^8^ Muscles and ligaments do not insert at the ends of the bones (where the cartilage is), but at several locations along the bone. Consequently, bones like the femur^9^ and the vertebrae^4^ are partially loaded under bending moments, which is reflected in their curved trabecular architecture. Finally, bone not only exists as compressive rods, like in the extremities, but also as bent, flat or humped structures, like ribs, skull, and vertebrae; they are strongly interconnected and not the isolated “floating” rods as described in tensegrity (Figure 1). Despite these differences, the musculoskeletal system has characteristics of a minimum‐weight structure and it depends on tensile elements for stability and integrity; hence, the musculoskeletal system can be called as a *tensegrity‐like* structure.

**FIGURE 2:**
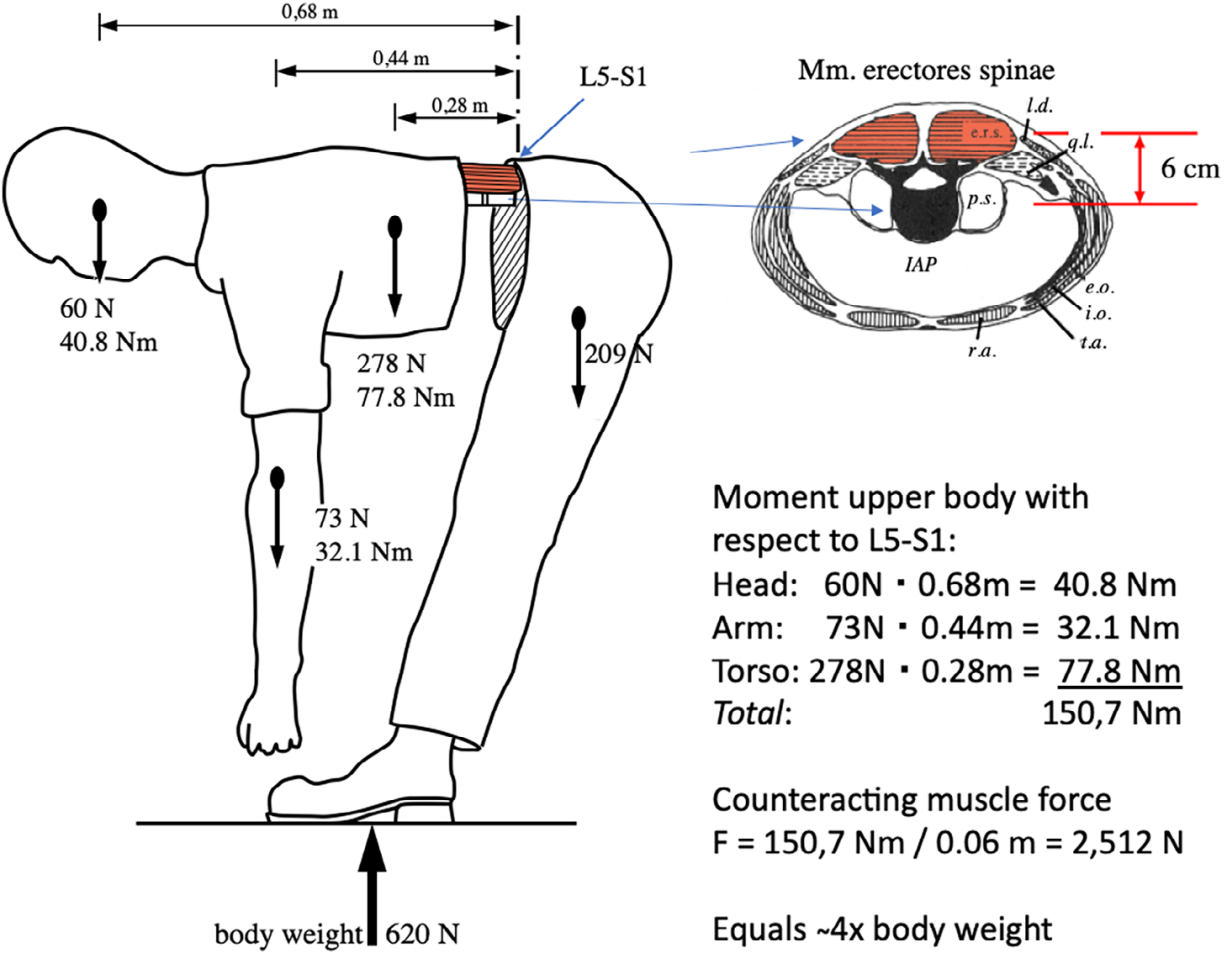
Muscular and gravity forces working on the body (62 kg) and the lumbar spine. In the situation drawn, the head, trunk and arms have a relatively large lever arm with respect to the joint center at L5‐S1. By contrast, the Mm. erectores spinae that counterbalance the resulting moment have a lever arm of only 6 cm. In the example drawn, the muscular force adds up to 2512 N, about four times body weight. Figure adapted from Grieve D, Phaesant S (1982): Biomechanics (Chapter 3). In: Singleton, WT, ed. *Body at Work—Biological Ergonomics*. Cambridge University Press, p.165

Adolescent idiopathic scoliosis (AIS) is a slow, three‐dimensional deformation of the spine.^10^ Structural deformations are caused by forces and the key question thus is: what forces induce AIS? In principle, there are three sources of loading on the human musculoskeletal system: muscle, gravity, and growth. Muscles are active elements and provide motion and stability to the human body in the gravitational field. Muscles are positioned in parallel and in close vicinity to the bones, which means that they have small lever arms and must deliver large forces to control balance and motion. As a result, skeletal loads may reach several times body weight, both in the limbs and in the spine (Figure 2).^11^, ^12^ Gravity is an external force: it is relatively small, but generally has a large lever arm to the center of rotation (Figure 2). Severe muscle atrophy^13^ and bone loss^14^ in astronauts after substantial time in space indicate that gravity is an existential force for terrestrial vertebrates: when absent, muscles and bone are unloaded and therefore atrophy. Skeletal growth, finally, is a slow, but particularly critical force for AIS, because scoliotic deformations coincide with the adolescent growth spurt. The growth of bone and cartilage enhances tension in muscles, ligaments and fascia and in fact guides their growth.

In AIS patients, the delicate balance between skeletal growth and the maturation of stabilizing tensile structures appears to be disturbed. Earlier, we showed with a physical model that differential strain between an extending spine and nonstretchable ligaments results in slow three‐dimensional deformations similar to AIS.^15^ Here we take a mechanobiological perspective in an attempt to identify the origin of stresses in the scoliotic spine. Understanding the etio‐pathogenesis of AIS will allow early recognition of risk factors and the development of therapies that prevent severe deformities and drastic treatments at later stages of AIS.

## 2 ANOTHER SCAR OF EVOLUTION?

In 1951, the anthropologist Wilton M. Krogman published *The Scars of Human Evolution*.^16^ In this landmark paper, he articulated the popular opinion that humans created a “terrific mechanical imbalance” by changing to bipedal locomotion and that they pay the price with back pain, ankle sprains, tedious child delivery, and jaws too small for wisdom teeth. While this opinion can be nuanced,^17^, ^18^ one may consider to add AIS as another scar of evolution, since among mammals it is only observed in humans.^19^ Bipedal gait was already common in dinosaurs and is still present in birds,^20^ but human bipedalism has changed the mechanics of the musculoskeletal system in a number of ways. First, it places the center of gravity above the pelvis. This substantially reduces the force needed to balance the head and upper body, which is reflected by a more than 50% reduction of bone density compared to those of large quadruped species, along with a similar loss of compression strength of the vertebrae.^21^ Furthermore, intervertebral disc height in humans is about twice as high as in animals of similar size, both in absolute and relative numbers.^22^ This may be an evolutionary advantage to permit a greater range of motion, but it also implies a larger potential for swelling and growth.

Another effect of bipedal posture is the development of spinal curvatures in the sagittal plane. The thoracic kyphosis is typical for mammals and also humans are born with a kyphotic spine.^23^ The cervical and lumbar lordosis are thought to develop after birth through dorsal muscles that lift the head and the trunk, respectively. This notion is supported by the observation that the lumbar curvature increases with the volume of the Mm. erectores spinae.^24^ The fully upright position of the human spine also implies lower anterior shear forces than in quadruped and nonhuman bipedal spines and are counterbalanced by the facet joints.^19^, ^25^ Certain regions of the thoracic and lumbar spine may even be subject to posterior shear loads, which reduces the contact force of the facet joints and therefore induces rotational instability.^26^ The unique, fully upright bipedal human posture thus is a risk factor for AIS and may indeed be considered as another scar of evolution. This is further supported by observations that experimental rats and mice without front legs have a stronger tendency to develop scoliosis than quadrupedal controls.^27^, ^28^

## 3 SPINAL BUCKLING, IS NOT IT?

**FIGURE 3:**
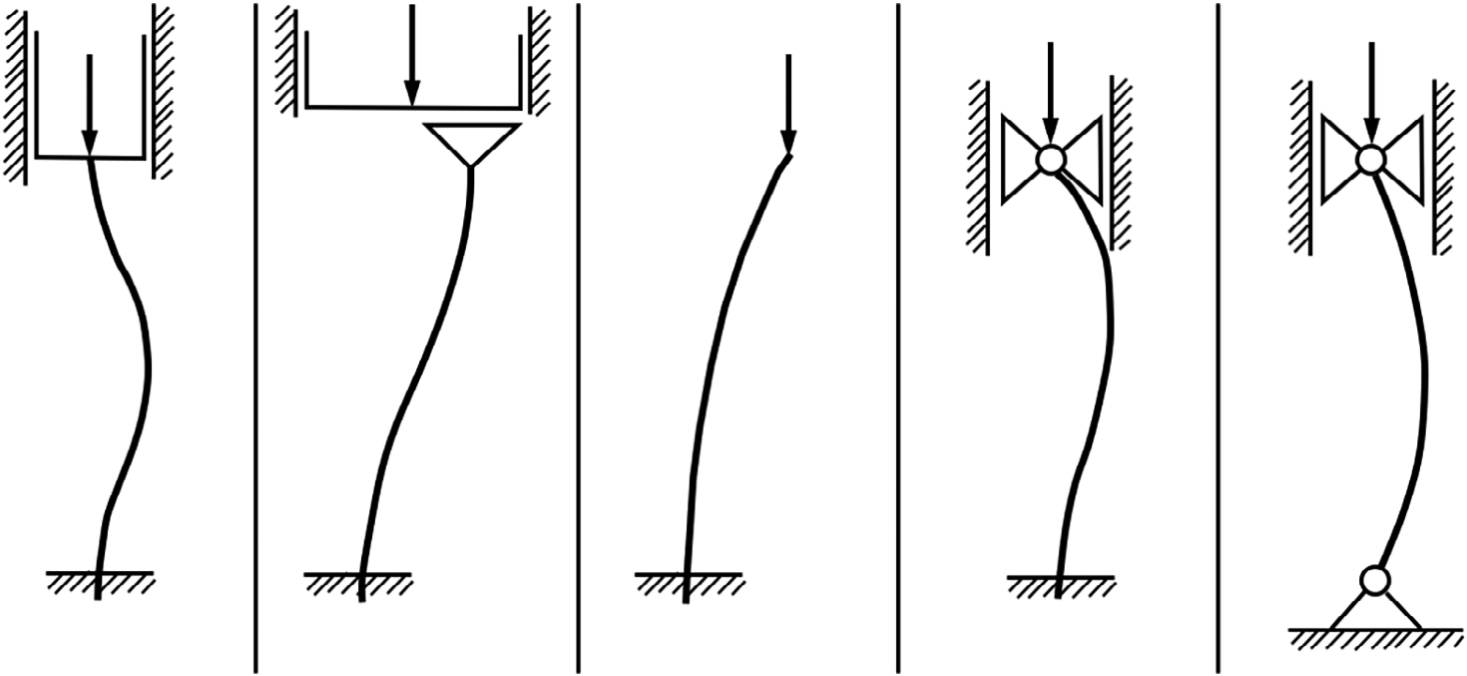
Rods buckling under axial compression. The mode of buckling strongly depends on the boundary conditions at the ends of the slender rod

There is an interesting resemblance between scoliotic deformations and Euler buckling (Figure 3).^29^, ^30^, ^31^ It appears that Lenke types^32^ can be related to different buckling modes,^29^, ^31^ which begs a serious consideration from this perspective. It seems important to note that girls, who have a much higher risk of developing AIS, have more slender spines than boys.^33^, ^34^ As the intervertebral disc height is the same in healthy boys and girls (about 7.5 mm),^35^ the spines of the latter show more flexibility^36^ and thus have a higher risk of buckling. Intervertebral discs in scoliotic girls *and* boys were found to be higher than those in healthy controls (+24% and +19%, respectively) and their vertebral cross‐sectional areas were smaller (−10% and −14%, respectively).^35^ It may be argued that these effects are the consequence of AIS rather than its cause, which is a general problem in cross‐sectional studies comparing subjects with and without AIS. Nevertheless, spines at risk for AIS tend to be more slender and thus more flexible, thereby lending credibility to the concept of buckling.

There are, however, several concerns that need to be raised. First, Euler buckling is an *instantaneous* mechanical instability of a straight elastic rod compressed at its ends. AIS, by contrast, is a slow process of spinal deformation that takes months or even years to develop. Second, the healthy vertebrate spine is not an elastic rod, but a series of vertebrae hinged by intervertebral discs. Each of the vertebral segments has a range of motion, including a neutral zone of several degrees in which it can bend without appreciable resistance.^37^, ^38^ The range of motion of the healthy spine by far exceeds the deformations that occur during the activities of daily life, so the vertebral segments are generally positioned within their neutral zone.^25^ Indeed, the ligamentous human spine (ie, the spine devoid of its musculature) is very unstable under compression with a measured buckling strength of only 21 N,^39^ less than the weight of the head. The spine thus needs to be stabilized and this is done by the tension of muscles, that together with bone, ligaments, and fascia form a tensegrity‐like system. The thorax^40^ and the facet joints^4^ support the vertebrae and also have a stabilizing effect on the spine. Concluding, there appears to be little use in considering the spine as an isolated, elastic rod that buckles under axial compression, at least for the onset of AIS in the young and healthy spine.

## 4 THE GROWING SPINE

The preadult musculoskeletal system is a growing *tensegrity‐like* construct. This implies that both the compressive bones and the tensile muscles and ligaments increase in length, while maintaining tension and integrity. The skeleton is leading and grows in the growth plates and the intervertebral discs. Growth is achieved by chondrocytes that divide in the direction of loading and subsequently hypertrophy to their regular size.^41^, ^42^ Once mature, the chondrocytes produce proteoglycans and collagens to form the extracellular matrix, which attracts and binds water through osmosis. Intervertebral discs grow until (about) the age of 12, after which their height essentially remains the same.^43^ This is related to the presence of notochordal cells up to the age of 13^44^; notochordal cells secrete growth factors and up‐regulate proteoglycan expression in intervertebral disc chondrocytes.^45^ These subsequently produce proteoglycans and collagen that form the highly osmotic extracellular matrix.^46^ Spinal growth thus essentially occurs through the hypertrophy of notochordal and chondrocyte‐like cells and osmotic swelling of the matrix they produce; these are slow, but very strong forces, commensurate with the deformations observed in AIS.

As the skeleton grows, the muscles, tendons, ligaments, and fascia are subjected to increasing tension. Muscles respond by the deposition of new sarcomere units at the ends of the muscle fibers (*sarcomerogenesis*),^47^ which lowers the stress and brings the fibers back to their optimal operating length.^48^ Tendon, ligament, and fascia, however, are much stiffer than muscle^49^ and thus more restrictive. Collagen fibrils are aligned in parallel, separated by a matrix of noncollagenous (nonfibrillar) components that cross‐link the fibers for mechanical integrity.^50^ During growth, the collagen fibrils slide past one another over the entire length of the tendon,^51^ which indicates a mechanism of reversible fiber‐to‐fiber bonding^52^; this secures high mechanical stiffness required for tensegrity of the musculoskeletal system.

## 5 DIFFERENTIAL GROWTH

**FIGURE 4:**
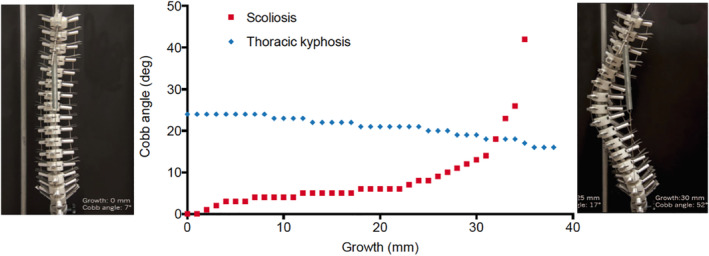
Differential growth in a physical spine model. The distance between the vertebrae is expanded while the metal wires representing the ligaments restrict this. We see a gradual flattening of the thoracic kyphosis (blue dots) and an induced scoliosis which starts slowly and increases exponentially after reaching instability (red dots). Left an anterior view on the original position of the spine with a Cobb angle of 7°, right the situation at the end of the experiment. Note that the vertebrae also show substantial axial rotation, For further details, see Crijns et al^15^

AIS is a slowly progressing, permanent deformation of the spine in an apparently healthy, growing child. Such deformation is reminiscent of *differential growth*, a mechanical phenomenon also found in the folding of a flower,^53^ the cortical brain,^54^ the gut,^55^ and the developing heart.^56^ It essentially occurs through a mismatch between the elongation of two tissues attached to each other and results in bending and torsion. Crijns and colleagues used a physical model of a growing thoracolumbar spine to show that an impeded elongation of the tendons leads to internal compression of the spine, which first straightens and then slowly warps out of the sagittal plane by lateral bending and ‐inevitably^57^‐ axial rotation (Figure 4).^15^ It should be emphasized that no external load was applied to the spine model, which implies that scoliotic deformations do not result from classical Euler buckling. Instead, there is a mismatch in growth of the spine and the connecting wires, resulting in internal stresses and a three‐dimensional, scoliotic deformation. The question is why this happens in some adolescents, but only in a small minority of mankind.

## 6 SKELETAL GROWTH

Skeletal growth is predominantly regulated by hormones, including Growth Hormone (GH), Insulin‐like Growth Factor (IGF), and sex hormones like estradiol.^58^ However, growth is also a force and thus can be modulated by mechanical stress. This phenomenon is described by the *Hueter‐Volkmann Law* ^59^, which states that increased compression acting on a growth plate retards bone growth and, conversely, reduced compression or tension accelerates it. In the context of AIS, the Hueter‐Volkmann Law is thought to underlie the wedge‐shaped deformation of the vertebrae in late stages of AIS,^60^ presumably a secondary effect of the scoliotic asymmetries.^61^ However, the principle also applies to linear bone growth, that is: without malformations, as shown experimentally in long bones of various species.^62^

It is well documented that AIS patients are in general taller (2‐4 cm in girls; 2‐6 cm in boys)^63^; show later (3‐6 months) but faster skeletal growth (2.9 vs 1.2 cm/year)^64^; have lower body mass index (BMI; 8‐11%)^65^; and have lower bone density (about −12% at 15 years of age)^66^, ^67^ than age‐matched controls. Low bone density is a very strong indicator of reduced muscular strength,^68^ as reported in osteoporotic patients^69^ and in bipedal humans, who have 48% to 60% lower spinal bone density than quadrupeds of similar size and weight.^21^ Bone density has been related to genetic, endocrine, hormonal and nutritional factors, but from a mechanical perspective (*use it or lose it*) the observation that AIS patients have lower bone density suggests that they experience less mechanical loading than age‐matched controls. This may be due to reduced muscle mass (−3.5%)^70^, ^71^ and muscle strength (11%^72^‐42%^73^), which in turn may be the result of deviating hormone levels^70^, ^74^ or other mechanisms. It is interesting to note that enhanced skeletal growth does not only apply to the spine, but also to the extremities: arm span and ulna and radius length, for example, are reported to be good predictors of AIS.^64^ Thus, AIS patients generally have reduced or delayed muscle mass, or in other words: a mismatch between skeletal growth and musculo‐ligamental maturation. Following Hueter‐Volkmann Law, reduced muscle strength results in decreased skeletal loading and therefore accelerated bone and cartilage growth, which indeed is widely reported in literature.^35^, ^63^, ^64^, ^75^

## 7 THE SWELLING INTERVERTEBRAL DISC

Another effect of reduced spinal compression is an increased height of the intervertebral discs. This is a well‐known physiological phenomenon in humans after a night of sleep,^76^ and is observed *in extremis* in astronauts who stay in space for several weeks or months and show an increase of spinal length up to 7 cm.^77^ The increase in disc height results from an osmosis‐induced fluid flow into the nucleus pulposus upon the release of mechanical compression.^46^ In AIS, the intervertebral disc height is much increased as compared to age‐matched controls^35^: Ponrartana and colleagues report an averaged 9.06 ± 0.85 vs 7.31 ± 0.60 mm in girls and 9.09 ± 0.87 vs 7.61 ± 1.00 mm in boys, a difference of 1.7 and 1.5 mm (24% and 19%), respectively. By comparison, a diurnal change of healthy disc height in humans is in the order of 0.5 mm (6.7%) per disc.^78^ Also Brink et al^79^ report that the elongation of the anterior thoracic spine of AIS patients is located in the discs, rather than in the vertebral bodies.

A swollen intervertebral disc points at a high osmotic pressure, a reduced mechanical compression, or both.^80^ As argued above, AIS patients generally have low muscle strength and reduced spinal loading. At the same time, the pressure in the nucleus pulposus is much higher in AIS patients (in the order of 0.25 MPa)^81^, ^82^ than in non‐AIS back patients and healthy persons in comparable postures (0.12‐0.15 MPa).^83^, ^84^ It is also reported that intervertebral discs of adult AIS patients still contain notochordal cells,^85^ while they normally disappear before the age of 13.^44^ A recent study links the presence of notochordal cells in the nucleus pulposus to mechanical loading.^86^ Li and colleagues observed in an *ex vivo* bioreactor study that the application of high‐amplitude dynamic compression on porcine discs results in more apoptotic cells, a catabolic gene expression profile, and a decreased GAG and collagen type‐II content, compared to groups with low‐amplitude dynamic loads or static compression. Dynamic compression results from the activities of daily life and it is essential for the viability of chondrocytes in cartilaginous tissues,^87^ because it drives interstitial fluid flow.^88^ Much external loading, however, deforms the matrix and cells and decreases the viability of notochordal cells^86^; this may explain the observed loss of notochordal cells in the growing child.^44^ Reversely, the presence of notochordal cells in the discs of adult AIS patients may indicate a reduced dynamic loading regime, which is commensurate with a reduced muscular activity or strength and a reduced bone density. Thus, reduced muscle mass results in reduced spinal loading, saves the notochordal cells in the nucleus pulposus and contributes to an increased intradiscal pressure and spinal growth. This essentially represents a positive feedback loop of disc pressure and progressive deformity as suggested earlier.^81^

## 8 ANNULUS FIBROSUS AND LIGAMENTS

**FIGURE 5:**
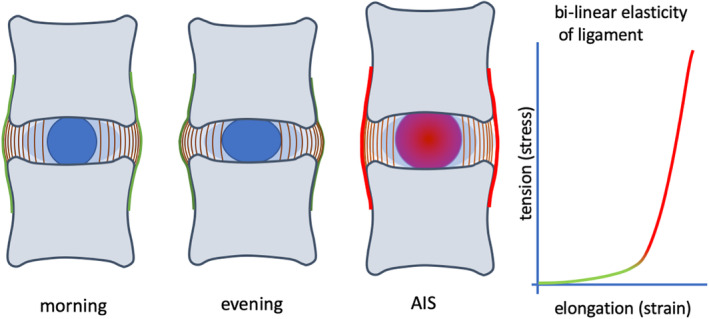
Tension in the ligaments and annulus fibrosus. A, Intervertebral disc height diurnal changes from morning (left) to evening (middle). In the case of AIS, intervertebral disc height is strongly increased by increased disc pressure, counterbalanced with higher tension in ligaments and annulus fibrosus (right). B, Typical bi‐linear elasticity of ligaments, with low stiffness in the toe‐region (green) and high stiffness in the strained region (red)

Hydrostatic and osmotic pressure can only exist in a confined environment. The nucleus pulposus is contained between the cartilage endplates of the adjacent vertebrae, and surrounded by the multi‐laminar annulus fibrosus. The vertebral bodies enclosing the intervertebral disc are further connected by the anterior and posterior longitudinal ligaments, which allow small deformations of the intervertebral discs, but limit excessive flexion and extension.^89^ During postnatal growth, the ligaments and the annulus fibrosus grow with the increasing pressure of the nucleus pulposus as discussed earlier. It is therefore interesting to observe that intervertebral disc height is increased from about 7.5 to 9.1 mm in AIS patients,^79^, ^90^ but that growth is effectively zero after the age of 12.^43^ This indicates that the swelling of the intervertebral disc is limited, presumably because the stretching potential of the annulus fibrosus and/or the anterior and posterior longitudinal ligaments is exhausted (Figure 5). Limited expansion is in fact a prerequisite for the increasing internal pressures reported by Meir and colleagues.^81^, ^82^ It appears, however, that collagen remodeling is strongly strain‐dependent,^91^ in the sense that enzymatic breakdown decreases with more tension and has a minimum close to zero at about 20% strain.^92^ This implies that ligaments that are under higher tension are less amenable to growth. Thus, while the pressure in the nucleus pulposus increases, the potential for remodeling and growth of the annulus fibrosus and ligaments is reduced. This results in an internal spinal stress that may be the driver of the scoliotic deformations, similar to the expanding screws in the physical spine model that are restrained by the metal wires.^15^

The question then rises why the annulus fibrosus and the ligaments do no longer grow with the increasing pressure of the nucleus pulposus. One possible reason for this is tissue maturation, which results in a stronger cross‐linking of the collagen fibers. Dahners and colleagues^93^ report that tension enhances ligament length in immature, but not in mature rabbits. In other studies, Dahners and colleagues report about agents that interfere with collagen fibril cross‐linking, including gentamycin, the polycation NKISK and relaxin.^52^ Of these, relaxin may be particularly relevant, because it is a sex hormone that modulates collagen and is upregulated after ovulation. A delay of menarche by 3 to 6 months, as observed in girls with severe AIS^64^ thus could come with low levels of relaxin and therefore increased cross‐linking that inhibits growth. It is further interesting to note the relationship between mechanical loading of the ligaments and the upregulation of relaxin^94^; this suggests that normal activities of daily life enhance remodeling and growth, while a lack of dynamic loading reduces growth capacity. This is commensurate with the observed reduction of muscle mass^70^ and muscular strength in AIS patients.^71^

## 9 DISCUSSION

### 9.1 The mechanobiological mechanism

The human body is a *tensegrity‐like* structure, in which the skeleton obtains its integrity by the tension of muscles, ligaments, and fasciae. As the child grows, bones and cartilage elongate the muscles, ligaments, and fascia and induce their remodeling and growth.^47^, ^50^ In order to accommodate the increasing body mass and lever arms, muscles must not only elongate, but also strengthen. In AIS patients, this balance between skeletal growth and muscular maturation appears to be disturbed. Considering the facts presented in literature and summarized above, the following scenario for the etio‐pathogenesis of AIS is suggested. During the growth spurt, there seems to be a delay in muscular maturation,^70^, ^71^ resulting in decreased tensegrity and a lowered prestress in the skeleton. According to *Hueter‐Volkmann* ^59^, reduced axial compression enhances the growth of bones, which is indeed observed in AIS patients.^64^, ^95^ Furthermore, decreased spinal compression results in a lower vertebral bone density^66^, ^67^, ^96^ and increased disc height.^8^, ^35^, ^79^ Reduced dynamic loading also allows the presence of notochordal cells in the nucleus pulposus,^85^, ^86^ which stimulate the production of proteoglycans and the assembly of the osmotic extracellular matrix.^97^, ^98^ The intradiscal pressure increases the intervertebral disc height to its limits,^43^ rendering the longitudinal ligaments and the annulus fibrosus under large tension (Figure 5). Under these conditions, ligaments can no longer remodel^92^ and therefore no longer grow with the intervertebral discs. The enhanced intradiscal pressure and locked ligamental remodeling embody the differential growth of the spine that results in scoliotic bending and rotation (Figure 4).^15^ The presented scenario provides a mechanical pathway for the slow development of the three‐dimensional spinal deformations so typical for AIS.

### 9.2 Paradox

**FIGURE 6:**
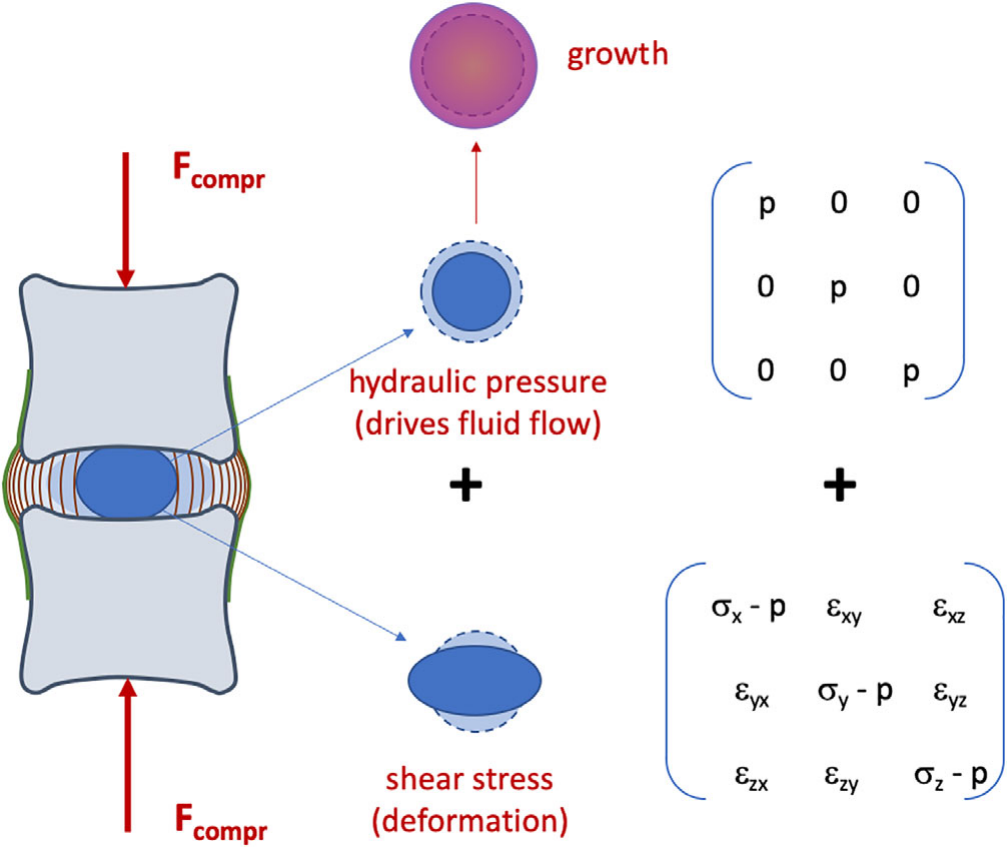
Spinal compression and stresses in the intervertebral disc. The spine is mainly loaded under axial compression (*F*_compr_), which results in a deformation and a stress in the intervertebral disc. Stresses can mathematically be divided into two components: an all‐sided pressure p which reduces the volume and remains shape; and a distortional shear stress which maintains the volume of the matrix but changes shape. Hydraulic stress drives the fluid flow which is necessary for the transport of nutrients and waste products and secures the vitality of the cells. Notochordal cells and chondrocytes thrive under hydrostatic pressure and respond by proliferation and matrix production, leading to growth. Shear stress, by contrast, induces katabolic and inflammatory gene expression, leading to apoptosis and matrix breakdown. The balance between hydraulic and shear stress determines cellular response and growth of the intervertebral disc

The differential growth hypothesis presents an interesting paradox, because it assumes a reduced muscular compression of the spine that results in higher intradiscal pressure. This seems to be at odds with studies in a loaded disc culture system, which showed an almost linear relationship between axial compression of a spinal segment and intradiscal pressure.^8^ The paradox is solved by the notion that interstitial growth of the nucleus pulposus, so critical in the onset and development of AIS, is also a stress. Notochordal cells and chondrocytes in the intervertebral disc thrive by hydrostatic pressure and react by proliferation and production of a high‐osmotic matrix. This matrix attracts and binds water and further raises intradiscal pressure (Figure 6). This pressure is balanced not by muscular forces on the spine, but by the annulus fibrosus and the longitudinal ligaments (Figure 5). If the increase in intradiscal pressure is slow, the annulus fibrosus and the ligaments have time to grow through sliding collagen fibers and detaching‐re‐attaching crosslinkers. Growth of the annulus fibrosus and the ligaments releases the intradiscal pressure. If the tension on the annulus and ligaments is too high, however, their remodeling is locked and no growth is possible.^92^ (such locking may also be due to a lack of relaxin as a result of late first menarche,^64^ and a subsequent increase of collagenous cross‐linking.) As a consequence of locking, the intradiscal pressure increases, which in turn favors the physiology of chondrocytes and notochordal cells and increases the production of proteoglycans, and so on in a vicious circle. External (muscular) loading not only increases hydrostatic pressure,^8^ but also induces deformation of the disc and the cells within (Figure 6). Such shear strain negatively affects the functionality of chondrocytes and notochordal cells, induce apoptosis^86^ and hence, a reduction of hydrostatic pressure. If intradiscal pressure is high, though, the deformability of the disc is low and the vitality of chondrocytes and notochordal cells is unaffected.

### 9.3 Limitations and other issues

Differential growth has been suggested as a physical mechanism for the onset and development of AIS.^15^ The current perspective paper identifies intradiscal pressure as the driver of this process, together with an inhibited growth of the longitudinal ligaments of the spine. While reduced muscular activity may underlie expansion of the intervertebral disc, the hypothesis does not explain the underlying cause of muscular weakness, which may have to do with hormone levels, physical activity and/or late menarche. The hypothesis also does not address secondary effects of AIS, like vertebral wedging^61^, ^99^ or muscular asymmetry.^100^ It further can be observed that AIS presents itself in different curve patterns, generally referred to as Lenke types.^32^ The mechanobiological mechanism suggested here (Figures 5 and 6) applies to the single intervertebral disc, not at the entire spine. It seems unlikely, however, that intradiscal pressure increases equally along the spine. This is confirmed by Tomaschevsky,^101^ who identified short straight spinal segments in an otherwise flexed spine in children who develop AIS later on. Alternatively, it can be argued that a spine with inflated intervertebral discs eventually stiffens and behaves like a curved elastic rod. Pasha recently showed in a finite element model that the primary sagittal curvatures of the spine determine the eventual scoliotic deformations seen in Lenke 1‐6.^31^, ^102^ This provides an attractive model for the explanation of the various curve patterns beyond the differential growth hypothesis.

While the focus of the paper was on AIS, there also exist other scoliotic deformations, like neuromuscular, congenital, or degenerative scoliosis. The latter appears to underlie a very different mechanism, since in de novo scoliosis the intervertebral discs are degenerated and therefore have a decreased intradiscal pressure which leads to lumbar spinal instability.^103^ Neuromuscular and congenital scoliosis, however, have much in common with AIS, because both come with substantially lower muscular activity than usual in healthy adolescents. This may lead to faster spinal growth due to lack of axial compression, and also a lock‐in of ligamental growth, resulting in differential growth. This effect may be obscured because neuromuscular scoliosis often involves muscular asymmetry, which can inherently contribute to skeletal deformities.

### 9.4 Muscular deficiency

It is an old tenet that AIS only occurs in humans. This may be due to the vertical position of the spine, which requires less muscular activity for stabilization and induces destabilizing posterior shear loads.^19^ It may also relate to the adolescent growth spurt, which is unique to humans.^104^ Indeed, no scoliosis has been observed in quadrupeds or nonhuman bipeds other than induced by trauma, surgery or specific mutations. One reason could be that spinal compression is intrinsically higher in quadrupeds and nonhuman bipeds,^25^, ^105^ as evidenced by higher bone density.^21^ Following *Hueter‐Volkmann*, axial growth in quadrupeds can be increased by a reduction of compression and thus by both, lower muscular strength and a more vertical position of the trunk. Machida^27^ and Liu^28^ showed that bipedal, upright standing rats and mice indeed are at higher risk to develop scoliosis compared to their native controls. In both studies, this risk was further increased by lowering the level of melatonin, which is known to have a relation to sleep,^106^ but also affects muscular function.^107^ Muscular deficiency thus appears to be a pivotal mechanical factor in the onset and development of AIS.

### 9.5 Hormones

While reduced muscular tension thus may underlie the onset and development of AIS, the sketched scenario does not explain the origin of such deficit. The higher prevalence of AIS among girls^108^ is strongly suggestive for a role of hormones, in particular sex hormones estradiol and testosterone. Estradiol is generally higher in girls, testosterone in boys.^109^ However, literature seems as yet inconclusive^110^, ^111^ as testosterone levels have been reported to be increased, but also decreased in AIS patients.^110^ Sleep hormone melatonin appears to have a direct effect on muscular function^107^ as well as the vertebral growth plates^112^ and has also been related to AIS in bipedal rats and mice.^27^, ^28^ The data regarding human melatonin levels are however mixed and its deficiency cannot be confirmed to play a role in the onset of AIS in humans.^113^ Another interesting hormone in relation to muscular strength is leptin, which inhibits hunger and is strongly correlated to lower muscle mass and body fat in AIS patients.^70^, ^74^ Ghrelin, another “hunger hormone,” is also reported to increase the risk of AIS.^74^, ^114^ How the upregulation of these hormones leads to muscular deficit, however, remains to be elucidated. Generally, hormones play complex roles in biology and have multiple and interacting functions. Absolute levels may vary during the day as well as during pubertal growth and their function strongly depends on the availability of the respective receptors. It appears unlikely, therefore, that one single hormone will prove to be responsible for the onset or development of AIS, or can be used for its treatment.

### 9.6 Animal models

An important drawback in the research on AIS is the lack of relevant animal models. Quadrupeds do not show idiopathic scoliosis, presumably because axial compression is higher^25^, ^105^ and because quadruped spines have a higher rotational stiffness.^115^ Scoliosis can only be induced in quadrupeds through drastic surgery, which then is not idiopathic by definition. An entirely different family of scoliosis models is found in teleosts: pinealectomy in salmon, for example, was observed to result in a similar spinal deformation as pinealectomy in the chicken.^116^ This implies that gravity is not a decisive factor in the onset and development of scoliosis. Gorman et al defined an experimental model with a strong genetic component, the guppy *curveback* syndrome.^117^ As in humans, scoliotic curvature in *curveback* develops after birth and does not progress after skeletal maturity; also, there is a female bias for the most severe deformations. While teleost models open interesting opportunities for investigating genetic factors in AIS,^118^ there is also an important anatomical difference: the lack of an intervertebral disc. Among vertebrates, only mammals have an intervertebral disc with a nucleus pulposus,^119^ which can be traced back to the embryonic development of the spine.^120^ Considering the pivotal role of hydraulic pressure in the nucleus pulposus as driver of AIS, there appears to be a different mechanical pathway for scoliosis in teleosts; supposedly, increased muscular compression exceeds buckling strength of the teleost spine.

### 9.7 Screening for early onset

The observation that scoliosis is the end stage of differential growth (Figure 4) begs the question whether predictors or earlier signs of AIS can be identified. Indeed, screenings among school children have been performed^121^, ^122^ but the need and efficacy of screening for AIS remains debated.^123^ It appears that early signs should be found in the sagittal plane, rather than the frontal plane, which only can detect lateral deformations and therefore late AIS. Given the observed deviations in AIS patients,^35^, ^79^ intervertebral disc height could be an interesting target. Tomaschewski^101^ studied the sagittal thoracic curvature under flexion in 686 children 9 to 10 year old. In 16.5% of them, she found a short vertebral segment that was straight and could not be flexed forward actively or passively. Of these 16.5%, 27% went on to develop idiopathic scoliosis within 1 year. A similar study was performed by Nakakohji^124^ who observed a severely decreased range of spinal flexion in 87 out of 93 AIS patients, while the 40 healthy controls could flex smoothly. The impaired forward flexion points at a limited elongation potential of the posterior ligaments, a situation that may be due to a strongly increased disc height and overstrained longitudinal ligaments as discussed above; unfortunately, such data were not provided in these studies. Other relevant predictors of AIS may be factors that contribute to muscular strength and skeletal remodeling, like hormones and physical activities during daily life.

### 9.8 Testing the hypothesis

The mechanobiology of differential growth in the adolescent spine is presented as an interaction of increased intervertebral pressure and locked ligamental growth. Both aspects can be tested in vitro in a loaded organ culture system. Entire intervertebral discs can be cultured under well‐defined mechanical and biochemical conditions, thereby controlling medium composition as well as static and dynamic spinal compression. While it has been shown that high dynamic pressure induces apoptosis in chondrocytes and notochordal cells,^86^ it may be hypothesized that high static pressure in the NP, which could be simulated by placing the IVD in a low‐osmotic medium, will rescue the cells and enhance matrix production and intradiscal pressure. Ligaments and other collagenous structures can also be placed in a bioreactor under static and dynamic tension,^93^ similar to tendon.^125^ Quasi‐static tension at various speeds may confirm the studies by Ghazanfari et al^92^ that axial tension decreases the elongation potential of ligaments and fasciae. It may further be hypothesized that dynamic loading enhances remodeling and thus growth of the constructs, while increased cross‐linking of the collagens would lock their elongation. The in vitro setting of bioreactors also allows interfering with ligamental cross‐linking by the addition of relaxin, NKISK, or gentamycin.^52^ Due to the limited availability of young human spines, it will be difficult to test the differential growth hypothesis on a thoracolumbar spine in vitro. When available, however, one can simulate growth by placing the spine in low‐osmotic medium and one can mimic ligamental locking by the application of stiff tension bands along the spine. Such studies are less likely to succeed with animal spines, because these have thinner discs and thus lower swelling capacity.

### 9.9 Prevention and treatment studies

The differential growth hypothesis suggests that high intradiscal pressure is the driver for the onset and development of AIS. Since AIS only occurs in humans and no relevant animal models exist, the hypothesis can only be tested in clinical studies. The most direct intervention would be the release of osmotic pressure in highly swollen intervertebral discs. This could be achieved in a controlled way by the injection of a low dose of Chondroitinase ABC, which induces an immediate breakdown of proteoglycans and ‐thus‐ a release of intradiscal pressure.^126^ Long‐term follow‐up studies in goats have shown that Chondroitinase ABC has a temporary degrading effect and that disc height loss (and thus intradiscal pressure) stabilizes after about 3 months.^127^, ^128^ Obviously, the injection of an enzyme in the intervertebral disc is an invasive procedure and there may be a long‐term risk of disc degeneration. Therefore, this procedure may run into ethical constraints. On the other hand, percutaneous injections in a day‐care clinic are less inconvenient than several months of bracing^129^ and indeed much less invasive than internal fixation.^130^

There are also indirect ways of releasing intradiscal pressure in young adolescents. One line of thinking is that insufficient muscular strength could be the inducer of enhanced spinal growth. Core stability training then would be helpful for loading the spine, thereby suppressing the presence and activity of notochordal cells in the nucleus pulposus.^86^ Better core stability also would increase the tensegrity of the spine and thereby reduce the risk of scoliotic deformities. Studies addressing core stability have actually been performed and proven to be quite effective, at least in young female AIS patients^131^ and somewhat older male patients.^132^ Core stability therefore also would be an interesting ‐albeit laborious‐parameter for early screening and prevention of AIS. Another line of thinking would be to decrease intradiscal pressure by stretching the locked ligaments. There are various conservative therapies that focus on stretching and flexibility of the spine,^133^ but none of them relates to the condition of the intervertebral disc. Yet stretching exercises could be beneficial both for releasing ligaments and for dynamic loading of the intervertebral disc. Various studies show beneficial effects of Schroth therapy in young but advanced AIS patients.^134^, ^135^ Overall, a beneficial effect on the onset and progress of AIS thus may be expected from gym classes at elementary school.

### 9.10 Summary and conclusion

Etio‐pathogenesis of AIS has been intensely studied in the past by many groups all over the world, yet the deformations observed remain essentially idiopathic. Many factors have been incriminated, but none of them appears to be exclusive or predictive. Most studies present a correlation rather than a physical mechanism (more particular: a force) that leads to the deformations observed. The suggested differential growth is a mechanism often observed in Nature and fits well with the timing and speed of scoliotic deformations during the adolescent growth spurt. It was already shown in a physical model of the spine that differential growth can lead to scoliotic deformations.^15^ The current perspective paper relates this model to well‐documented observations in AIS patients and controls, including muscular mass, bone density, and disc height. This mechanobiological perspective also suggests physical cues for AIS, including low muscular mass and increased intradiscal pressure; this may be helpful for designing prevention and treatment strategies for AIS.

## CONFLICT OF INTEREST

The author has no conflict of interest to disclose.
